# Imputation of genotypes in Danish purebred and two-way crossbred pigs using low-density panels

**DOI:** 10.1186/s12711-015-0134-4

**Published:** 2015-06-30

**Authors:** Tao Xiang, Peipei Ma, Tage Ostersen, Andres Legarra, Ole F Christensen

**Affiliations:** Center for Quantitative Genetics and Genomics, Department of Molecular Biology and Genetics, Aarhus University, Tjele, DK-8830 Denmark; INRA, UR1388 GenPhySE, CS-52627, Castanet-Tolosan, F-31326 France; Pig Research Centre, Danish Agricultural and Food Council, Copenhagen, DK-1609 Denmark

## Abstract

**Background:**

Genotype imputation is commonly used as an initial step in genomic selection since the accuracy of genomic selection does not decline if accurately imputed genotypes are used instead of actual genotypes but for a lower cost. Performance of imputation has rarely been investigated in crossbred animals and, in particular, in pigs. The extent and pattern of linkage disequilibrium differ in crossbred versus purebred animals, which may impact the performance of imputation. In this study, first we compared different scenarios of imputation from 5 K to 8 K single nucleotide polymorphisms (SNPs) in genotyped Danish Landrace and Yorkshire and crossbred Landrace-Yorkshire datasets and, second, we compared imputation from 8 K to 60 K SNPs in genotyped purebred and simulated crossbred datasets. All imputations were done using software Beagle version 3.3.2. Then, we investigated the reasons that could explain the differences observed.

**Results:**

Genotype imputation performs as well in crossbred animals as in purebred animals when both parental breeds are included in the reference population. When the size of the reference population is very large, it is not necessary to use a reference population that combines the two breeds to impute the genotypes of purebred animals because a within-breed reference population can provide a very high level of imputation accuracy (correct rate ≥ 0.99, correlation ≥ 0.95). However, to ensure that similar imputation accuracies are obtained for crossbred animals, a reference population that combines both parental purebred animals is required. Imputation accuracies are higher when a larger proportion of haplotypes are shared between the reference population and the validation (imputed) populations.

**Conclusions:**

The results from both real data and pedigree-based simulated data demonstrate that genotype imputation from low-density panels to medium-density panels is highly accurate in both purebred and crossbred pigs. In crossbred pigs, combining the parental purebred animals in the reference population is necessary to obtain high imputation accuracy.

**Electronic supplementary material:**

The online version of this article (doi:10.1186/s12711-015-0134-4) contains supplementary material, which is available to authorized users.

## Background

Implementation of genomic selection (GS) [1] in breeding programs requires dense molecular marker genotypes since increasing marker density increases the probability that a marker is in strong linkage disequilibrium (LD) with a quantitative trait locus (QTL) [2]. However, the high costs of genotyping are a key constraint to efficient implementation of GS [3]. To partly overcome this problem, it has become current practice to genotype candidates for selection using low-density single nucleotide polymorphism (SNP) chips (up to 10 000 SNPs), while a limited number of individuals chosen as reference animals are genotyped with a high-density chip (50 000 SNPs or more). Imputation is then carried out from low density to high density [4, 5]. Studies on US Jersey cattle have confirmed that the accuracy of GS does not decline when using imputed genotypes if the low-density panel includes more than 3000 evenly distributed SNPs [4]. Furthermore, missing genotypes that are not called by some of the standard genotyping methods must be imputed prior to inclusion in models for GS [6]. Overall, genotype imputation is generally considered as an initial step for GS.

Genomic selection has been successfully applied for purebred populations [7, 8], but it is also possible to select purebred animals for crossbred performance by combining information from crossbred animals with genomic information from purebred animals [9]. Crossbreeding is very common in pigs bred for meat production because of the increased performance of crossbred compared to purebred individuals [10]. Due to the difficulty and high cost of collecting phenotypic and pedigree data on crossbred animals [11] and genotyping costs, data on both purebred and crossbred animals are rarely available. Performances of GS in crossbred and purebred pigs may differ because of dominance effects in combination with different allele frequencies in the two pure breeds, and because the extent of LD between SNPs and QTL may differ between crossbred and purebred populations. Thus, the effects of SNPs may be breed-specific [9].

Algorithms for genotype imputation (such as that implemented in Beagle [12]) depend crucially on LD patterns across markers [13], which may be breed-specific. Therefore, the performance of genotype imputation might differ between crossbreds and purebreds. Since genotypes are rarely available for crossbred individuals in livestock, most studies that have investigated the critical factors that affect the performance of imputation have been based on purebred plant [14] and livestock populations [15–18]. Recently, an analysis of imputation from 6 K to 50 K SNP chip genotypes in crossbred taurine beef cattle was reported [19], but, to our knowledge, this has not been evaluated in crossbred pigs.

In this study, different scenarios of imputation from lower density (5 K) to higher density (8 K) SNP chips were compared using two Danish pig breeds, Landrace and Yorkshire, and a two-way crossbred Landrace-Yorkshire population. Differences in imputation accuracies between purebred and crossbred animals were investigated to set up an optimal strategy for imputation from a low-density (8 K) to a medium-density (60 K) SNP chip in crossbred pigs and results were validated using a simulated dataset of crossbred medium-density (60 K) genotypes. Previous studies indicated that the relationship between imputed and reference individuals is one of the major factors that affects performance of imputation [3, 6, 20]; Hayes et al. [3] reported that it could account for up to 64 % of the variation in accuracy of imputation in sheep. Thus, to better understand the results in the current study, we tried to quantify relationships between animals within and across datasets, using genomic relationships and indexes of haplotype similarities.

## Methods

### Animals and genotypes

All data were provided by the Danish Pig Research Centre. The numbers of genotyped purebred Danish Landrace (LL), Danish Yorkshire (YY) and two-way crossbred Danish Landrace-Yorkshire pigs were 9328, 9393 and 5639, respectively. Crossbred animals that had a Landrace sire and a Yorkshire dam were referred to as ‘Landrace_Yorkshire’, while those that had a Yorkshire sire and a Landrace dam were referred to as ‘Yorkshire_Landrace’. Crossbred animals consisted of 4432 Landrace_Yorkshire (LY) and 1207 Yorkshire_Landrace (YL) pigs. Purebred and crossbred animals were born between 1998 and 2013 and 2009 and 2012, respectively. All crossbred pigs were results of matings between the two pure breeds. Pedigrees of both purebred and crossbred pigs were available and all crossbred animals could be traced back to their purebred ancestors. Among the 5639 crossbred pigs, 4956 had genotyped sires (n = 1580) but only nine pigs had genotyped dams (n = 4). In addition, 1441 maternal grandsires of the crossbreds were genotyped. Crossbred animals were divided into two subsets: those that had a genotyped sire (4956) and those that did not (683).

Both pure breeds were genotyped with the Illumina PorcineSNP60 Genotyping BeadChip [21]. Two different versions of the 60 K SNP chip (Illumina PorcineSNP60 v1 and PorcineSNP60 v2) were used to genotype purebred animals, i.e. about 50 % animals with each version. About 2 % of the SNPs worked in one version but not in the other version and vice versa. The two different chip versions should be taken into account when applying a quality filter on SNPs. Previous unpublished analyses (Tage Ostersen, Danish Pig Research Centre, personal communication) on purebred pigs showed that when applying a quality filter on SNPs, varying the minimum call rate for individuals from 70 to 90 % did not affect the accuracy of genomic predictions significantly. This combined with the fact that very few animals had a call rate between 80 % and 90 %, we chose to set the minimum call rate of individuals to 80 %. SNP quality controls were applied for the dataset that consisted of both pure breeds combined as follows: SNPs with a call rate less than 90 % were removed; SNPs with a minor allele frequency lower than 0.01 across both purebred populations were removed; SNPs that showed a strong deviation from Hardy Weinberg equilibrium within breeds (p < 10^−7^) were also excluded. After filtering, a common set of 42 483 SNPs was retained for the two purebred populations (these are referred to as 60 K). Crossbred individuals were genotyped with a 8.5 K GGP-Porcine Low Density Illumina Bead SNP chip [22] and very few animals had a call rate between 80 % and 90 %. Using the same quality controls for the crossbred animals as for purebred animals (except for Hardy-Weinberg equilibrium, which does not hold for crossbred animals), 7940 markers were retained, which represents a subset of the 42 483 SNPs retained for the purebred animals. SNPs were mapped to pig chromosomes using the pig genome build 10.2 [23].

### Imputation scenarios

To mimic an imputation strategy similar to what is routinely applied in real genetic evaluations, 5162 LL and 5130 YY pigs that were born in 2012 and 2013 were used as validation animals. The remaining 4166 LL and 4263 YY pigs that were born before 2012 were used as reference animals for imputation. All 5639 crossbred pigs were treated as validation animals. Based on pedigree information, the parents of the crossbred animals were all born before 2012. Thus, if the parental genotypes of the crossbred individuals were known, they were included in the reference population.

To compare the performance of imputation between purebred and crossbred animals, first imputation from 5 K to 8 K was evaluated, which was applied to the common set of 7940 SNPs. SNPs were sorted by map position and then, one of every three SNPs was masked (i.e. 2647 SNPs were masked) and the remaining SNPs were retained to represent the lower density panel (5 K). To ensure consistency of imputation results, this was repeated three times by shifting the masked SNPs by one position each time. For the purebred populations, imputations were first done by using one of the pure breeds as reference population, which consisted of individuals that were either from their own breed (within-breed scenario) or the other pure breed (external-breed scenario), i.e., we imputed Landrace animals using Yorkshire animals as the reference population and vice versa. Then, each breed was imputed by a combined Landrace and Yorkshire population (combined-breed scenario). Finally, for the crossbred population, imputation was done by using either a single purebred reference population (one of the two pure breeds) or a combined Landrace and Yorkshire population (4166 LL + 4263 YY). In order to eliminate the effect of population size of the reference panel, its size was fixed to 8429 animals for all scenarios of imputation of crossbred animals. Thus, when only one purebred reference population was used, it had to also contain animals that were born after 2011 in order to constitute such a large population of genotyped single purebred animals.

A second strategy of imputation from 8 K to 60 K was implemented in purebred animals by using a combined reference population. In the validation dataset, SNPs that were not present on the low-density chip were masked and subsequently imputed. However, results of imputation from 5 K to 8 K for both purebred and crossbred animals, and those of imputation from 8 K to 60 K for purebred animals could not completely describe how imputation worked from 8 K to 60 K for crossbred animals. Therefore, the quality of imputation from 8 K to 60 K for crossbred animals was validated using simulated data from the 60 K SNP chip for crossbred animals. Genotypes of crossbred animals were simulated according to the genotypes of their ancestors based on frequencies of recombination according to Haldane’s mapping function [24]. Additional file 1 [see Additional file 1] describes in more detail the steps used to simulate the 60 K genotypes for 5639 crossbred animals. All imputations were done using the software Beagle version 3.3.2 [12].

### Evaluation of imputation accuracies

Accuracies of imputation for each strategy are presented by mean correct rates and mean correlation coefficients between imputed genotypes and real genotypes. Mean correct rates were calculated per SNP (across individuals) as the proportion of correctly imputed genotypes, and then averaged over all imputed SNPs (for details, see [25]). Correlation coefficients were calculated per SNP across all imputed individuals and then averaged over SNPs, following [26].

### Genomic relationships across breeds

Genomic relationships among individuals were estimated based on 8 K real genotypes using VanRaden’s method [27] as $\mathbf{G} = \frac{\mathbf{Z}{\mathbf{Z}}^{\hbox{'}}}{2{\displaystyle \sum }p\left(1-p\right)},$ Where **Z** is a matrix of genotypes coded as {−1, 0, 1}, and p was set to 0.5, so that a unique reference point was chosen and results could be compared within and across breeds. Compared to pedigree-based relationships, all estimated genomic relationships will be biased upwards, but bias will be the same across breeds and subgroups of animals. The genomic relationships are thereby comparable both across and within breeds, which is the objective of our study. For each individual in the validation population, the average genomic relationship to individuals in the reference population was computed by averaging coefficients from the appropriate section of the genomic relationship matrix. Furthermore, for each crossbred individual in the validation population, the average of the top10 relationships between this individual and individuals in the reference population [28] was also computed. To visualize the distribution of relationships, density curves of genomic relationships were drawn. In addition, as suggested by [29], a principal components analysis (PCA) of the matrix of genomic relationships was conducted for a preliminary analysis of the genotypes, since PCA can help to investigate ethnic background of individuals [30].

### Proportion of shared haplotypes between reference and validation populations

Following imputation by Beagle, 8 K phased genotypes were available for all animals in the reference and validation populations. It was assumed that a haplotype consisted of a specific number of consecutive SNP alleles in the same phase. Lengths of haplotypes were set to 10, 20, 30, 50 and 100 SNPs. If a haplotype in the validation population could exactly match at least one haplotype at the same position in the reference population, this haplotype was considered to be shared between the reference and validation populations. The number of shared haplotypes was counted and then divided by the total number of haplotypes in the validation population, and this was referred to as the proportion of shared haplotypes (PSH). In addition, the number of unique haplotypes (NUH) in the reference populations was counted to represent the number of different patterns for a specific haplotype length across all individuals in the reference population. Values for PSH and NUH were averaged over non-overlapping windows of a specific size.

## Results

### Imputation strategy ‘5 K to 8 K’

#### Performance of purebred imputation

**Fig. 1:**
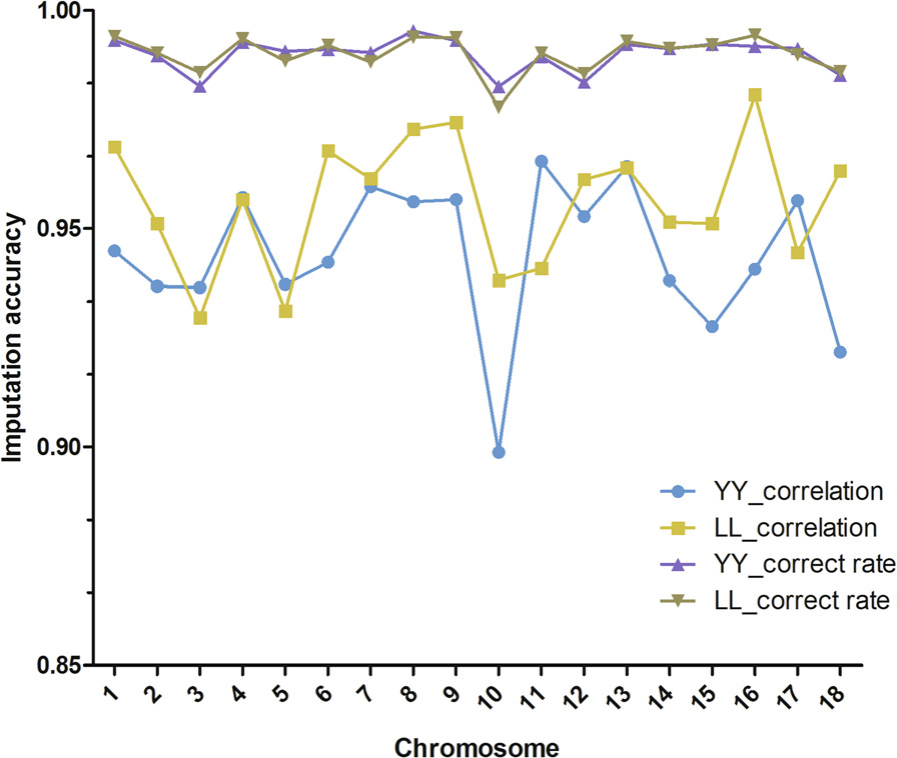
Variation in imputation accuracy for the scenario from 5 k to 8 k across different chromosomes using within-breed reference populations. Within-breed reference means Landrace pigs were imputed using a reference population that consisted of Landrace pigs only and Yorkshire pigs were imputed using a reference population that consisted of Yorkshire pigs only

Figure 1 shows imputation accuracies from 5 K to 8 K across the 18 autosomes for the purebred Landrace and Yorkshire pigs when using a within-breed reference population. On the whole, accuracies did not vary much between chromosomes. Correct rates were larger than or equal to 0.99, except for chromosomes 3, 10, 12 and 18 for both breeds. No differences in mean correct rate were observed between the two purebreds. Correlation coefficients between imputed and true genotypes ranged from 0.90 (chromosome 10) to 0.97 (chromosome 13) for the Yorkshire breed and from 0.93 (chromosome 3) to 0.98 (chromosome 16) for the Landrace breed. Slight differences in mean correlation coefficients (0.012) were observed between the two breeds. Overall, the Landrace breed performed slightly better than the Yorkshire breed, especially in terms of the correlation coefficients. Variations of correlation coefficients were generally consistent with those of correct rates across the whole genome.

**Fig. 2:**
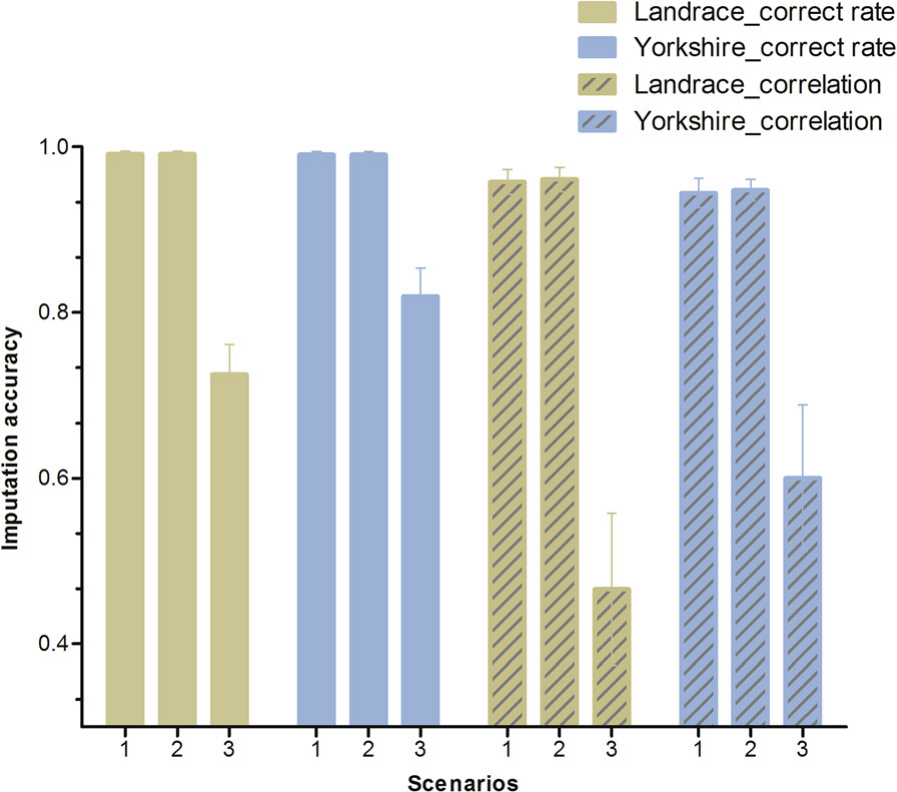
Comparison of imputation accuracies obtained by different imputation scenarios in Landrace and Yorkshire breeds. 1 indicates that the reference population consisted of either 4166 LL or 4263 YY, depending on the respective breed (within-breed scenario); 2 indicates that the reference population consisted of 8429 combined LL and YY (combined-breed scenario) and 3 indicates that the reference population consisted of animals that belonged to another purebred breed (external-breed scenario), which means that Landrace animals were imputed using a reference population that contained Yorkshire pigs only and Yorkshire animals were imputed using a reference population that contained Landrace pigs only. Error bars are standard deviations

Comparison of imputation accuracies that were obtained in the different imputation scenarios from 5 K to 8 K for purebred animals is in Fig. 2. Correct rates for purebred animals were identical for the within-breed and combined-breed scenarios for both breeds, but correlation coefficients increased slightly (around 0.01) in the combined-breed scenario. However, in the external-breed scenario, both correct rates and correlation coefficients decreased sharply for both breeds compared with the within-breed scenario. Landrace animals had marked lower imputation accuracies than Yorkshire animals in the external-breed scenario, whereas imputation accuracies were similar between the two breeds in the within-breed and combined-breed scenarios, both in terms of correct rates and correlation coefficients.

#### Performance of imputation for crossbred animals and comparison with that of purebred animals

Table 1 summarizes the performance of imputation from 5 K to 8 K for purebred and crossbred animals when the size of the reference populations was fixed to 8249. When a combined reference population was used, imputation was better for purebred animals than for crossbred animals in terms of correct rate, although the improvement was very small (around 0.006). However, in terms of correlation coefficient, imputation accuracy was slightly greater for crossbred animals than for Yorkshire pigs, but slightly lower for crossbred animals than for Landrace pigs. However, if the reference population used for imputation of crossbred animals was replaced by a pure breed population, both correct rate and correlation coefficient decreased dramatically by about 0.10 and 0.25, respectively. Imputation of crossbred animals using a reference population that included only Yorkshire pigs resulted in a larger decline in accuracies than using a reference population that included Landrace pigs only. Table 2 presents imputation accuracies (correlation coefficients) for the subsets of crossbreds with a genotyped sire and those with a non-genotyped sire. Regardless of the reference population used, the differences were small, although the subset of crossbreds with a genotyped sire always had slightly higher accuracies than the subset of crossbreds with a non-genotyped sire.

**Table 1:** Accuracy of imputation from 5 K to 8 K for Landrace (LL), Yorkshire (YY) and crossbred animals

Imputed	Reference	Correct rate	Correlation
LL	LL + YY	0.9910	0.9606
YY	LL + YY	0.9907	0.9477
Crossbred	LL + YY	0.9849	0.9566
Crossbred	LL	0.9034	0.7595
Crossbred	YY	0.8667	0.6871

**Table 2:** Imputation accuracy (correlation coefficients) from 5 K to 8 K for crossbred animals with genotyped and non-genotyped sires

Reference	Sire non-genotyped	Sire genotyped
LL + YY	0.9529	0.9576
LL	0.7596	0.7603
YY	0.6883	0.6911

The first row indicates the components of the reference population whether it consists of a purebred Landrace (LL), Yorkshire (YY) or a combined population (LL + YY). There are 4956 crossbred animals with genotyped sires and 683 with non-genotyped sires in each subset, respectively

#### Genomic relationships across breeds

**Fig. 3:**
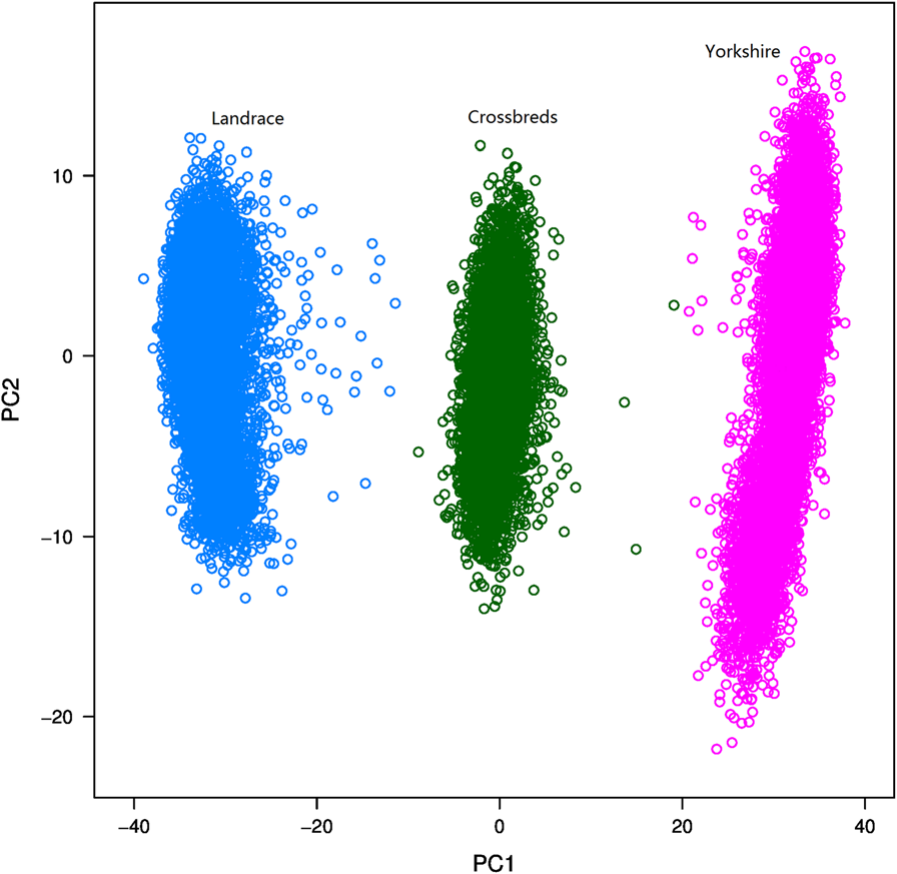
Principal components analysis on the matrix of genomic relationships within breeds. The first two main principal components are presented on the x-axis and y-axis, respectively. The proportions of variability across individuals explained by PC1 and PC2 were 22.92 % and 0.88 %, respectively

The two main principal components on the matrix of genomic relationships of each individual across Landrace, Yorkshire and crossbred Landrace-Yorkshire animals are in Fig. 3. The first two components explained 22.8 and 0.9 % of variability across individuals, respectively. The first principal component (x-axis) separated the three populations, whereas the second component (y-axis) could not distinguish between breeds. There was hardly any connection between the two clouds of points representing the Landrace and Yorkshire breeds, whereas the cloud of points representing the crossbred Landrace-Yorkshire population was generally in between. Connections between Landrace and crossbred pigs seemed to be slightly tighter than those between Yorkshire and crossbred pigs, since there are many more points distributed in the interval between Landrace and crossbred pigs than between Yorkshire and crossbred pigs. Overall, connections between crossbred and purebred animals were not strong.

**Fig. 4:**
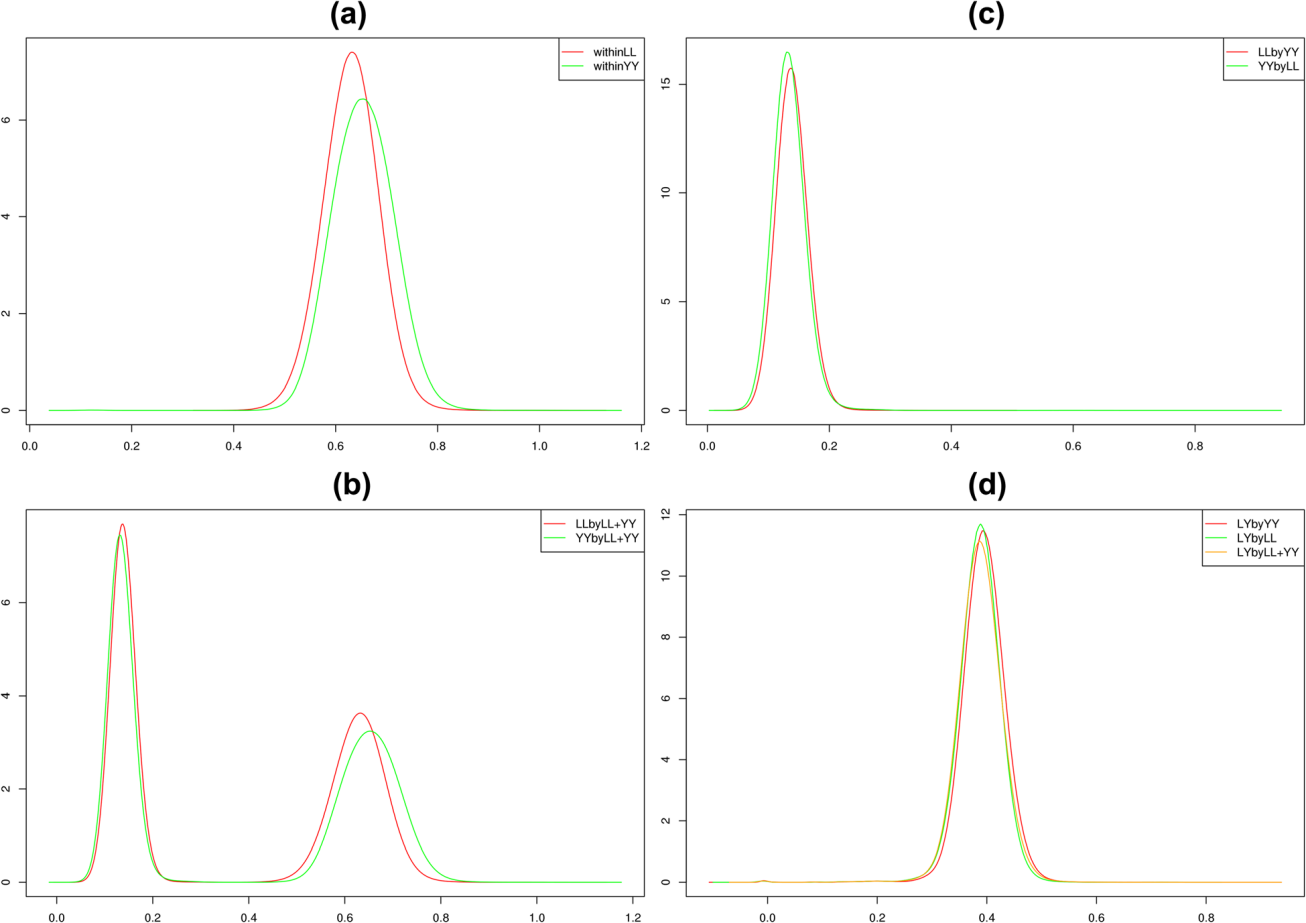
Density curves of genomic relationships between reference and validation populations for different imputation scenarios.(**a**) within-breed scenario for purebred Landrace and Yorkshire; (**b**) imputation of purebreds by using a combined Landrace and Yorkshire population; (**c**) the external-breed scenario for purebred Landrace (LLbyYY) and Yorkshire (YYbyLL) and (**d**) imputation of crossbreds by using either one purebred reference population (LYbyYY and LYbyLL) or a combined Landrace and Yorkshire population (LYbyLL + YY). All scenarios were under the imputation strategy of ‘5 K to 8 K’

**Fig. 5:**
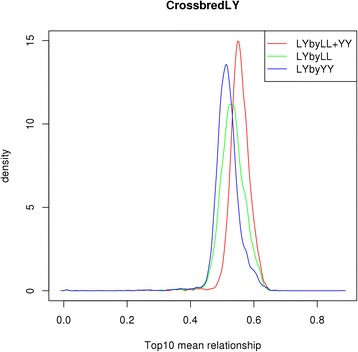
Density curves of the top 10 mean genomic relationships between crossbred animals and three different reference populations. The reference population consisted either of a single purebred reference (LYbyLL and LYbyYY) or a combined Landrace and Yorkshire population (LYbyLL + YY)

Table 3 provides averaged genomic relationships between individuals in the reference and validation populations that correspond to the different imputation scenarios evaluated. The results in Table 3 show that the mean relationship within breeds was always the largest for all scenarios. When a breed was imputed using a reference population that comprised individuals of the other pure breed (external-breed scenario), the mean relationship decreased to approximately one fifth of that obtained with the within-breed scenario. When a combined reference population was implemented to impute purebred animals, logically, mean relationships were intermediate to the values found with the within-breed and external-breed scenarios. In addition, regardless of which reference population was used to impute crossbred animals, mean relationships were similar. Distributions of genomic relationships between reference and validation populations obtained with different scenarios of imputation are represented by density curves in Fig. 4. In general, for the Landrace and Yorkshire purebred pigs, the distributions of relationships were similar regardless of which reference population was used (as shown in Fig. 4a, b and c). For the crossbred animals, density curves were highly consistent whether the reference population consisted of animals from one breed or from different populations (Fig. 4d). The density curves of the top10 mean genomic relationships between crossbred animals and animals from the three different reference populations are in Fig. 5. Landrace pigs had closer top 10 mean genomic relationships with crossbred animals than Yorkshire pigs, and by construction, animals of the combined-breed population had higher top10 mean genomic relationships with crossbred animals than either of the populations that consisted of a pure breed.

**Table 3:** Average genomic relationship between reference and validation populations

	LL	YY	LL + YY
LL	0.6398	0.1388	0.3874
YY	0.1343	0.6442	0.3932
Crossbred	0.3869	0.3943	0.3875

The first row indicates the components of the reference populations, whether it consists of a purebred breed Landrace (LL), Yorkshire (YY) or a combined population (LL + YY)

#### Proportions of shared haplotypes (PSH)

Proportions of haplotypes that were shared between reference and validation populations for different imputation scenarios are in Table 4. The results show that PSH decreased as the length of haplotypes increased. For purebred animals, PSH was always very similar between Landrace and Yorkshire breeds when a within-breed or a combined population was used as reference population, regardless of the length of the haplotypes. However, PSH decreased dramatically when the reference population consisted of only of the other breeds (external-breed). Differences in PSH existed between Landrace and Yorkshire breeds in different scenarios: for the within-breed scenario, LL had slightly higher PSH than YY when haplotypes were longer than 30 markers, but slightly lower PSH for shorter haplotypes; for the external-breed scenario, PSH was consistently lower for LL than for YY. Among the scenarios for imputation of crossbred animals, PSH was highest when a combined population was used as reference population. PSH declined when the reference population was changed from a combined population to a pure breed population. In particular, PSH was lowest when the reference population consisted of only the Yorkshire breed. The number of unique haplotypes (NUH) that existed in the reference population for different imputation scenarios is in Table 5, which shows that if only one breed was used as a reference population, Landrace animals always provided more haplotypes than Yorkshire animals. Furthermore, if the reference population consisted of a combined population, it always had a much larger NUH than if it consisted of only one breed. However, the NUH in the combined population was not equal to the sum of the NUH in each breed and was in fact smaller than this sum. In other words, some haplotypes were shared by the two breeds.

**Table 4:** Proportions of shared haplotypes between the reference and validation populations for different imputation scenarios

Validation	Reference	10^a^	20^a^	30^a^	50^a^	100^a^
LL	LL	0,9965	0,9814	0,9549	0,8838	0,6417
LL	YY	0,5043	0,1836	0,0877	0,0463	0,0141
LL	LL + YY	0,9972	0,9832	0,9556	0,8847	0,6606
YY	YY	0,9967	0,9817	0,9545	0,8825	0,6295
YY	LL	0,6806	0,3419	0,2232	0,1267	0,0364
YY	LL + YY	0.9971	0.9829	0.9589	0.8843	0.6579
Crossbred	LL	0,8579	0,6758	0,5947	0,5016	0,3280
Crossbred	YY	0,8108	0,6132	0,5125	0,4004	0,2765
Crossbred	LL + YY	0,9902	0,9606	0,9135	0,8092	0,5357

^a^Number of consecutive SNP alleles assumed for each haplotype. LL stands for Landrace; YY stands for Yorkshire. All the scenarios were under the imputation strategy of ‘5 K to 8 K’

**Table 5:** Numbers of unique haplotypes that existed in the reference populations for different imputation scenarios

Validation	Reference	Size of reference	10^a^	20^a^	30^a^	50^a^	100^a^
Purebred	LL	4166	63	223	441	956	2297
Purebred	YY	4263	58	216	445	966	2298
Purebred	LL + YY	8429	109	432	880	1916	4585
Crossbred	LL	8429	79	314	669	1579	4170
Crossbred	YY	8429	74	300	665	1571	4101
Crossbred	LL + YY	8429	109	432	880	1916	4585

^a^Number of consecutive SNP alleles assumed for each haplotype. LL stands for Landrace; YY stands for Yorkshire. All the scenarios were under the imputation strategy of ‘5 K to 8 K’. Numbers in the table are averages over non-overlapping windows of a specific size

### Imputation strategy ‘8 K to 60 K’

**Fig. 6:**
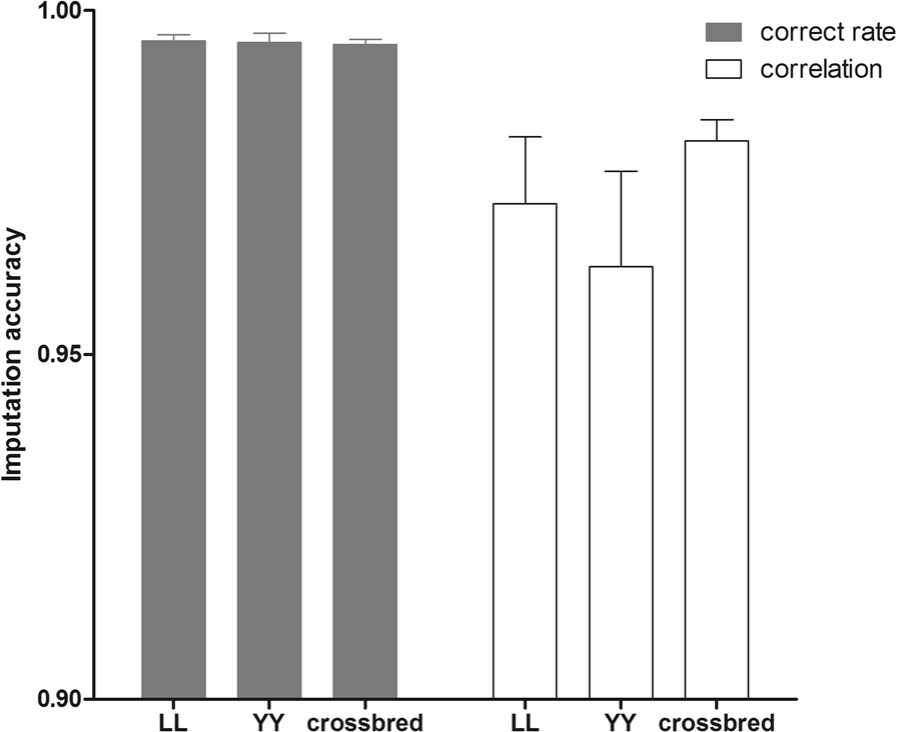
Comparison of imputation accuracies from 8 K to 60 K across breeds. Real genotypes were used for purebred Landrace and Yorkshire animals but simulated genotypes were used for crossbreds. Error bars are standard deviations

Figure 6 shows the comparison between imputation accuracies from 8 K to 60 K across breeds. The 60 K datasets comprised real genotypes for purebred animals and simulated genotypes for crossbred animals. According to Fig. 6, in terms of correct rate, performance of imputation for crossbred animals was almost as good as that for purebred animals. Fig. 6 also shows that crossbred animals performed even better than purebred animals in terms of correlation coefficients. Comparison of the results with the corresponding imputation scenarios in strategy ‘5 K to 8 K’ (first three lines in Table 1) clearly indicates that both correct rates and correlation coefficients are larger for the ‘8 K to 60 K’ strategy. For instance, accuracies of imputation from 8 K to 60 K for Landrace and Yorkshire pigs were about 0.005 and 0.015 larger than those from 5 K to 8 K for the correct rate and correlation coefficient, respectively. Before performing imputation from 8 K to 60 K in the simulated crossbred datasets, first we investigated the imputation from 5 K to 8 K in both the simulated and the real genotyped crossbred datasets. Results (not shown) showed that the performance of the simulated crossbred dataset was very close to that of the real crossbred dataset (0.004 greater correct rates).

## Discussion

Our aim was to verify the performance of imputation in Danish purebred and crossbred pigs using different scenarios. First, we studied imputation from 5 K to 8 K in genotyped purebred and crossbred datasets; the performance of imputation for each autosome of the purebred animals was evaluated only in the within-breed scenario; then imputations in purebred and crossbred animals were compared in within-breed, external-breed and combined-breed scenarios. Second, imputation from 8 K to 60 K was evaluated using genotyped purebred and simulated crossbred data. Overall, across all imputation scenarios, correct rates and correlation coefficients were consistent with each other, i.e. higher correct rates were associated with higher correlation coefficients.

The performance of imputation for purebred animals was high and consistent across the whole genome, which indicated that the strategy performed well for all pig autosomes. Among the 18 pig autosomes, imputation was, however, slightly worse on chromosomes 3, 10, 12 and 18, which is consistent with the results of a study on the average LD on pig autosomes using a similar dataset [31]. Among the pig autosomes, autosomes 10 and 12 had a relatively low average LD, which tends to decrease the length of shared haplotypes and therefore decreases imputation accuracy, since Beagle relies crucially on local LD structure [12]. Moreover, specific SNPs on a chromosome with an extremely low minor allele frequency (MAF) reduce the average correlation coefficient for the chromosome. For instance, three SNPs on chromosome 10 had an extremely low MAF (0.000097, 0.00039 and 0.00029, respectively) in the Yorkshire dataset. Correct rates for these three SNPs were 0.994, 0.997 and 0.998, but correlations coefficients were −0.0017, 0.00045 and −0.000027, respectively. When these three SNPs were removed, the correlation coefficient for chromosome 10 increased from 0.90 to 0.93. However, in the Landrace dataset, these SNPs had a MAF of 0.497, 0.185 and 0.499, respectively, and therefore they were retained in the analysis.

Based on Fig. 2, we concluded that pooling two purebred populations did not improve imputation accuracy compared to using a purebred reference population within a breed. This is in agreement with some previous studies in ruminants, which showed that combining reference populations from different breeds did not improve within-breed imputation [3, 20]. A possible explanation is that haplotypes on which imputation relies are less conserved across pig breeds compared to within breeds and those that were conserved were already present in the within-breed reference population. The sharp decrease in imputation accuracies when an external breed was used as reference population also supports that haplotypes are less conserved across breeds. However, several other studies [32, 33] showed that multi-breed reference populations enhance imputation accuracies compared to a single-breed reference population, but it should be noted that, in these studies, the within-breed reference population was small and imputation was done from high-density genotyping data to sequence data, which was not the case in our study. Therefore, to impute genotypes in purebred pigs, the reference population should include at least some individuals from the breed itself or a closely related population.

**Fig. 7:**
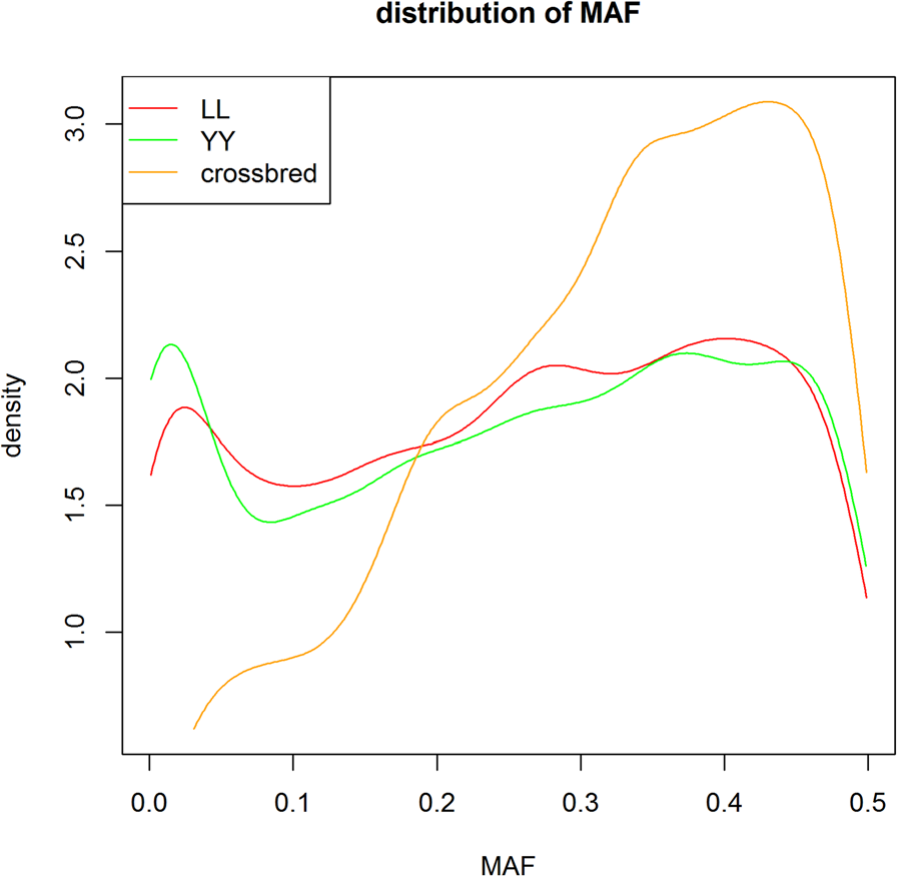
Density curves of minor allele frequency of the 2647 masked SNPs in the 8 K SNP chips. LL and YY represent Landrace and Yorkshire breeds, respectively. Allele frequency was calculated for each SNP across all individuals within the validation population

Based on Table 1, imputation in crossbred animals with a reference population that combined the two purebred populations performed almost as well as imputation in purebred animals, especially in terms of correlation coefficients. One possible explanation for crossbred animals having slightly greater correlation coefficients but lower correct rates compared to purebred animals may be due to the quality control criterion used (MAF > 0.01) across both purebred populations. The distribution of MAF of the masked SNPs in the imputation strategy ‘5 K to 8 K’ for Landrace (LL), Yorkshire (YY) and crossbred animals is in Fig. 7. This Figure shows that some SNPs had a MAF equal to 0 within a breed but not in crossbred animals. Crossbred animals tended to have higher MAF and SNPs with a very low MAF were more likely to occur for purebred animals, which decreases the correlation and increases the correct rate [6]. Imputation accuracies of crossbred animals significantly decreased when the reference population consisted of animals from only one breed. A previous study [3] suggested that imputation accuracies are expected to improve if sires and other ancestors were in the reference data, because relatives share common and longer stretches of haplotypes than distantly related animals [34]. In this study, up to 88 % of the sires of crossbred animals were present in the combined purebred reference population. Haplotypes of crossbred animals can be accurately identified and imputed based on the haplotypes of their relatives. Logically, crossbred animals that were imputed using a single breed reference population had much lower imputation accuracy. One explanation is that some haplotypes of the breed that is not in the reference population are not “detected” by the imputation software which, therefore, tries to impute them based on the other breed, which has a different LD pattern. In other words, by removing one breed from the reference population, all information from one parent and its ancestors is removed. This effect is visualized in Fig. 3, which shows that there were no connections between the two purebred populations for the first principal component (x-axis), and both breeds appeared to have almost equally weak connections with crossbred animals. Thus, both contributing pure breeds should be included in the reference population when imputing crossbreds to avoid inaccurately estimated haplotype blocks due to breed composition. In general, when imputing crossbred animals, it is desirable to include as many individuals of their purebred parental breeds in the reference population as possible.

Interestingly, Fig. 2 and Table 1 show that Landrace pigs had higher imputation accuracies than Yorkshire pigs when a reference population that consisted of a within-breed or a combined population was used, whereas Landrace pigs performed less well than Yorkshire pigs when the reference population consisted of an external breed. Among the factors that can affect imputation accuracies and were put forward by Iwata and Jannink [14], (genomic) relationships between the validation and reference populations constitute a major factor. In this study, the two pure breeds had similar family structures, which resulted in the distribution of genomic relationships between validation and reference populations being similar for the two breeds. As shown in Fig. 4a, b and c, there was no obvious difference in the density curves of relationship coefficients for Landrace and Yorkshire animals across different imputation scenarios. Thus, average genomic relationships between the validation and reference populations were similar for Landrace and Yorkshire pigs, as shown in the first two rows of Table 3. However, based on Table 3, it was not obvious that higher genomic relationships between the validation and reference populations would lead to higher imputation accuracies, as was proposed in many other studies, such as [3, 25]. Similarly, imputation accuracies for crossbred animals were also higher when imputation was done using a reference population of Landrace pigs only compared to Yorkshire pigs only, although the average genomic relationship between the crossbred validation population and the Landrace reference population was smaller than that between the crossbred validation population and the Yorkshire reference population, as shown in the last row of Table 3. All of these unexpected results indicate that the average genomic relationship is not sufficient to completely characterize the performance of imputation.

A possible explanation why imputation accuracies for crossbred animals were higher when imputation was done using a reference population of Landrace pigs only compared to Yorkshire pigs only is that close relationships play a much greater role in imputation accuracies than distant relationships [35]. According to Fig. 5, the density curves of the top10 mean genomic relationships suggested that crossbreds had a closer relatedness with Landrace pigs than with Yorkshire pigs. One fact is that the number of Landrace-Yorkshire crossbreds (4432) in the crossbred dataset was much larger than the number of Yorkshire-Landrace (1207) and most of the purebred sires were genotyped and included in the reference population. This fact may lead to improved performance of imputation of crossbred animals, which is consistent with the result that subsets with genotyped sires had slightly higher imputation accuracies than subsets with non-genotyped sires (Table 2). However, a closer examination of the results in Table 2 shows that the subset of non-genotyped sires resulted in a higher accuracy when imputation used a reference population that consisted of Landrace pigs only compared to Yorkshire pigs only and that it also resulted in a higher accuracy than the subset of genotyped sires when imputation used a reference population that consisted of Yorkshire pigs only. Thus, we conclude that having a genotyped sire is not the main cause of the differences in imputation accuracies for crossbred animals when imputation used a reference population that consisted of Landrace pigs only compared to Yorkshire pigs only. Another possible interpretation of why imputation accuracies for crossbred animals were higher when imputation used a reference population that consisted of Landrace pigs only compared to Yorkshire pigs only is that the Landrace breed contains Yorkshire haplotypes. The present Danish Landrace population is based on the old Danish Landrace breed, with some known imports from other European Landrace breeds in the 1970s. It is also known that imported Yorkshire animals were crossed with the original Danish Landrace stock in the 1890s, but it was later attempted to weed out these Yorkshire crosses again [36]. Thus, it is possible that the current Danish Landrace breed contains some Yorkshire haplotypes, but not vice versa. Finally, one remarkable difference between this study and other studies is that the size of the reference populations was much larger (10 to 20 times) in our study. A large number of reference animals can provide a large number of haplotype blocks and increase the possibility that specific haplotypes in the validation population match those in the reference population. When the reference population is very large, even a small proportion of close relationships can provide many shared haplotypes between reference and validation populations and thereby improve imputation accuracies.

The proportion of shared haplotypes can explain differences in performance of imputation among scenarios across breeds. A higher PSH indicates that a larger proportion of the haplotypes in the validation population, which need to be imputed, can be matched to corresponding haplotypes in the reference population and thereby be more accurately imputed. In general, our results agree with this hypothesis, as shown in Table 4. This could be one reason why imputation of a purebred or crossbred population by using a reference population that consists of Landrace animals only, always performed better than by using a Yorkshire reference population, although all other important factors (such as relationships, LD and MAF) were very similar in the two pure breeds. The fact that LL had slightly smaller PSH than YY, when the haplotypes were short (haplotype consisted of < 30 markers), but larger PSH when the haplotypes were long, indicates different patterns of sharing: long haplotypes are from recent ancestors and short haplotypes are from old ancestors, and there were more genotyped Landrace sires than genotyped Yorkshire sires. Table 5 quantitatively shows that although the combined-breed scenario provides more diverse haplotypes in the reference population than the single-breed scenario, these non-conserved haplotypes would not contribute to improve imputation of purebred animals. Clearly, the corresponding PSH in Table 4 did not increase as the reference population was changed from a within-breed to a combined population. Likewise, the simultaneous increase in PSH and NUH illustrates quantitatively the importance of using a reference population that consists of a combined population for the imputation of crossbred animals.

The higher accuracies of imputation obtained from 8 K to 60 K than from 5 K to 8 K for purebred animals confirmed previous studies [6], which showed that increasing the number of SNPs in low-density chips can improve the performance of imputation, because with denser SNPs local LD across markers becomes stronger. Therefore, it can be inferred that the performance of imputation for crossbred animals would also be marginally improved in the 8 K to 60 K scenario. Accuracies of imputation from 8 K to 60 K for purebred animals and simulated 60 K crossbreds were promising. To check that the simulation gave realistic results, the performance of imputation from 5 K to 8 K with a simulated crossbred dataset was compared with the performance of imputation from 5 K to 8 K with the real crossbred dataset (results not shown). The performance of imputation with the simulated 8 K dataset was slightly better than with the real 8 K dataset. The slight increase in accuracy was due to the simulation using haplotypes phased by Beagle. Thus, Beagle performed imputation based on data that had been generated under its own underlying model. Our results show that the improvement is negligible. Therefore, results from the simulated crossbred dataset can be trusted. It should be noted that there was an upper limit to the accuracy of phasing if the SNPs were sufficiently dense to be in high LD [12]. From an economic point of view, 8 K markers in a low-density panel seem sufficiently dense for imputation to medium-density (60 K) panels.

In pig breeding, imputation for purebred animals has also been done from very low densities (384 SNPs) to 60 K densities [37–39]. Consequently, we also evaluated the imputation accuracy from very low density (425 SNPs, 1 % of total SNPs retained) to 8 K in a crossbred dataset with a reference population that combined animals from both pure breeds. However, the accuracies were very low, around 0.7 and 0.5 for correct rates and correlation coefficients, respectively, which seems inadequate to implement genomic evaluation for crossbred performance in pigs.

Our goal was to compare the imputation performance between purebred and crossbred animals. We used the Beagle software. Although many other software programs have been developed for imputation, their comparison was beyond the scope of our study. All the imputation scenarios were executed on a Linux server with an Intel (R) Xeon (R) E5450@3.00 GHz CPU. The system is configured to allow computation with a maximum of four cores and a total of 32 GB RAM. Running time for imputing chromosome 1 of purebred animals in the within-breed and external-breed scenarios and strategy “5 K to 8 K” was 4 h ± 10 min, while the running time for imputing chromosome 1 of purebred animals in the combined-breed scenarios was around 6.5 h. The running time for imputing chromosome 1 of crossbred animals was about 6.5 h ± 15 min when different reference populations were used. For strategy “8 K to 60 K”, only the combined-breed scenario was implemented in purebred and crossbred animals and the running time for imputing chromosome 1 of crossbred animals was 67 h ± 30 min.

## Conclusions

Using the software Beagle, imputation performs very well and consistently across the whole genome and, as well, in crossbreds as in purebred animals, when the reference population combines animals from both parental breeds. For purebred animals, a reference population of within-breed animals ensures a good performance of imputation, especially when the size of the reference population is large. A combined reference population does not increase imputation accuracy for purebred animals compared to a within-breed reference population. A reference population that consists of an external breed only results in very poor imputation accuracy. For crossbred animals, a highly accurate imputed 60 K crossbred dataset can be achieved from 8 K by using a reference population that combines both parental breeds. The best method for imputation of crossbred animals is to include all purebred parental breeds in the reference population. Relationships can account for differences in imputation accuracy, but its effect will be limited by the size of the reference population. The proportion of shared haplotypes between the reference and validation populations gives an appropriate interpretation for the performance of imputation in both purebred and crossbred pigs.

## Additional file

Additional file 1:
**Pedigree-based simulation.** Description: The process to simulate a medium density (60 K) crossbred chip is described in which simulation uses real genotypes of purebred ancestors to simulate genotypes of their crossbred offspring.
