# Supplementary material for: Imputation of genotypes in Danish purebred and two-way crossbred pigs using low-density panels
Source: Genet Sel Evol. 2015 Jun 30;47(1):54. doi: 10.1186/s12711-015-0134-4 (PMC4486706; doi:10.1186/s12711-015-0134-4)
Supplement: Additional file 1: — Pedigree-based simulation. Description: The process to simulate a medium density (60 K) crossbred chip is described in which simulation uses real genotypes of purebred ancestors to simulate genotypes of their crossbred offspring. [file 12711_2015_134_MOESM1_ESM.docx]

Format: .docx

Title: Pedigree-based simulation.

Description: The process to simulate a medium density (60K) crossbred chip is described in which simulation uses real genotypes of purebred ancestors to simulate genotypes of their crossbred offspring.

In this additional file, we present the pedigree-based simulation process of creating a medium density (60K) crossbred chip data. Simulation used real genotypes of purebred ancestors to simulate genotypes of their crossbred offspring, as follows.

Landrace and Yorkshire can either be sires or dams of those crossbred pigs. To mimic the real data structure, the crossbred pigs were split into three main categories (see Table 1 and Table 2).

Category one was the situation when the parental genotypes of crossbreds were known, so that the crossbred progenies could be formed directly through a simulation algorithm. The simulation algorithm is an imitated meiosis process on the basis of a premise that SNPs were sorted by their physical positions on each chromosome already: according to Haldane's map function [[34](#_ENREF_34)], the number of crossovers between two phases within one chromosome follow a Poisson distribution, with parameter equal to the genetic distance of the chromosome in Morgan; the genetic distance was assumed such that markers separated by 10 kb of DNA had an expected rate of chromosomal crossovers of 0.01 per generation; it was assumed that the places where crossovers happened follow a uniform distribution along each chromosome; a ‘gamete’ of one individual would then be obtained through the meiosis process; each progeny would consist of such two dropped gametes, one from each parent. One crucial premise of this algorithm is awareness of the two phases of the parents. Software Beagle was used to phase genotypes in purebreds before we implemented the simulation meiosis process.

Category two was that sires of crossbreds were genotyped, but dams of crossbreds were not. Most crossbred individuals in the real dataset belonged to this category. In order to generate the genotypes of those dams, we simulated their genotypes from one generation earlier (maternal grandparents of crossbreds). If one of the grandparents’ genotypes were unknown, it would then be traced back to the previous generation (categories II-2 and II-3 in Table 1), and simulated in the same manner. This procedure would be processed until four generations back from crossbreds (category II-3 in Table 1). However, genotypes of some individuals in F_-4_ generation were still lacking (not included in reference panels). To keep the structure of dataset as analogous as a real dataset, we artificially created the genotypes of those individuals as follows: each chromosome for one individual was randomly sampled from that chromosome of another individual that was not included in its reference panel. These created individuals are shown within square brackets in Table 1 and Table 2.

Category three was a situation where neither sires nor dams of crossbreds were genotyped (Table 2). Simulation was done on those crossbreds’ paternal families as well as maternal families. For all the three main categories, progenies were generated based on the original mating order that was recorded in pedigree information, if the true genotypes of those purebreds were known. When the artificially created purebred individuals were involved in simulation process, we used random mating to generate progenies. Finally, 5,639 crossbreds, which consisted of 4,432 LY and 1,207 YL, were generated by the simulation algorithm.

For example, one crossbred YL (F_0_) from Category II-3 in Table 1. Its sire (F_-1__sire) was genotyped, but not the dam (F_-1__dam). One earlier generation (F_-2_) was traced back according to the pedigree information, which means we looked at the maternal grandfather (F_-2__Mgf) and maternal grandmother (F_-2__Mgm) of the crossbred YL. Animal F_-2__Mgf was genotyped, but not F_-2__Mgm. Another earlier generation (F_-3_) was needed to be traced back. Likewise, the great grandfather (F_-3__Ggf) of crossbred YL was genotyped, but not the great grandmother (F_-3__Ggm). Thus, one more previous generation (F_-4_) had to be traced backward. Only the father of great grandmother (F_-4__Fa_ggm) was genotyped, but not the mother of great grandmother (F_-4__Mo_ggm). The tracing was stopped at the F_-4_ generation, thus, we had to artificially create the genotypes of F_-4__Mo_ggm. For the created F_-4_ female Landrace, the first chromosome would be randomly selected from another Landrace individual’s chromosome one; then chromosome two was sampled from chromosome two of a third individual and so on until chromosome eighteen had been sampled. Then, based on the simulated meiosis algorithm, F_-4_ generation would produce the genotypes of F_-3__Ggm; F_-3__Ggm mated with F_-3__Ggf and they reproduced the F_-2__Mgm; alike, F_-2__Mgm mated with F_-2__Mgf and they reproduced the F_-1__dam; finally, F_-1__dam mated with F_-1__sire and the crossbred YL was obtained.

**Table 1 - Simulation process for crossbreds for which at least one parent are genotyped.**

Category one represents the situation that parental genotypes for crossbreds are known; category two stands for the situation that genotypes of sires for crossbreds are known, but not the dams. Mating pattern is the way of simulating progenies, either based on mating order that recorded in pedigree information or random mating. LL is Landrace; YY is Yorkshire; LY is crossbred Landrace-Yorkshire and YL is crossbred Yorkshire-Landrace. F0 _crossbreds is the crossbred generation; F-1 _sires and F-1 _dams represent the parental generation of crossbreds; F-2_Mgf and F-2_Mgm are maternal grandfather and maternal grandmother for crossbreds respectively; F-3_Ggf and F-3_Ggm are great grandfather and great grandmother for crossbreds, representing sires and dams of the F-2_Mgm respectively; F-4_Fa_ggm and F-4_Mo_ggm are the parents of F-3_Ggm. Numbers within round brackets ( ) stand for genotypes of that number of animals are unknown. Their genotypes were simulated, basing on their corresponding parental genotypes; numbers within square brackets [ ] represent genotypes of that number of animals were made according to the way that described in the main text; numbers without any brackets mean genotypes of that number of animals are known. Short dash (-) means this information was not used.

| Category | F_0__Crossbreds | F_-1__Sires | F_-1__Dams | F_-2__Mgf | F_-2__Mgm | F_-3__Ggf | F_-3__Ggm | F_-4__Fa_ggm | F_-4__Mo_ggm | Mating pattern |
| --- | --- | --- | --- | --- | --- | --- | --- | --- | --- | --- |
| I | 9 YL | 5 YY | 4 LL | - | - | - | - | - | - | in pedigree |
| II-1 | 92 LY | 58 LL | (53 YY) | 38 YY | 36 YY | - | - | - | - | in pedigree |
|  | 286 YL | 182 YY | (157 LL) | 65 LL | 47 YY | - | - | - | - | in pedigree |
| II-2 | 946 LY | 293 LL | (486 YY) | 170 YY | (216 YY) | 203 YY | 95 YY | - | - | in pedigree |
|  | 343 YL | 160 YY | (213 LL) | 117 LL | (137 LL) | 146 LL | 67 YY | - | - | in pedigree |
| II-3 | 2751 LY | 549 LL | (1441 YY) | 322 YY | (612 YY) | 203 YY | (309 YY) | 126 YY | 187 YY + [19 YY] | at random |
|  | 529 YL | 333 YY | (339 LL) | 245 LL | (234 LL) | 146 LL | (164 LL) | 85 LL | 38 LL + [83 LL] | at random |

**Table 2 - Simulation process for crossbreds for which genotypes of parents are unknown.**

Category III-1 is simulation process for crossbred Landrace-Yorkshire and category III-2 is simulation process for crossbred Yorkshire-Landrace. Mating pattern is the way of simulating progenies, either based on mating order that recorded in pedigree information or random mating. LL is Landrace; YY is Yorkshire; LY is crossbred Landrace-Yorkshire and YL is crossbred Yorkshire-Landrace. F0_crossbreds is the crossbred generation; F-1_sires and F-1_dams represent the parental generation of crossbreds; F-2_Gf and F-2_Gm are paternal or maternal grandfather and paternal or maternal grandmother for crossbreds respectively, decided by corresponding process; F-3_Ggf and F-3_Ggm are great grandfather and great grandmother for crossbreds, representing sires and dams of the F-2_Gm respectively; F-4_Fa_ggm and F-4_Mo_ggm are the parents of F-3_Ggm. Numbers within round brackets ( ) stand for genotypes of that number of animals are unknown. Their genotypes were simulated, basing on their corresponding parental genotypes; numbers within square brackets [ ] represent genotypes of that number of animals were made according to the way that described in the main text; numbers without any brackets mean genotypes of that number of animals are known. Short dash (-) mean the information was not used.

| Category | F_0__crossbreds | F_-1__Sires | F_-1__Dams | F_-2__Gf | F_-2__Gm | F_-3__Ggf | F_-3__Ggm | F_-4__Fa_ggf | F_-4__Mo_ggf | Mating pattern |
| --- | --- | --- | --- | --- | --- | --- | --- | --- | --- | --- |
| III-1 | 643 LY | (125 LL) | **-** | 48 LL | (32 LL) | 19 LL | 22 LL | - | - | in pedigree |
|  |  |  |  |  | (64 LL) | 44 LL | (59 LL) | 50 LL | 27 LL + [28 LL] | at random |
|  |  | **-** | (404 YY) | 152 YY | (58 YY) | 29 YY | 20 YY | - | - | in pedigree |
|  |  |  |  |  | (218 YY) | 99 YY | (145 YY) | 103 YY | 37 YY + [68 YY] | at random |
| III-2 | 40 YL | (29 YY) | **-** | 25 YY | (11 YY) | 9 YY | 10 YY | - | - | in pedigree |
|  |  |  |  |  | (13 YY) | 16 YY | (12 YY) | 11 YY | 6 YY + [6 YY] | at random |
|  |  | **-** | (32 LL) | 29 LL | (7 LL) | 6 LL | 7 LL | - | - | in pedigree |
|  |  |  |  |  | (21 LL) | 20 LL | (21 LL) | 20 LL | 11 LL + [10 LL] | at random |
